# Quantitative estimation of phospholipid molecules desorbed from a microbubble surface under ultrasound irradiation

**DOI:** 10.1038/s41598-023-40823-0

**Published:** 2023-08-22

**Authors:** Reina Kobayashi, Jun Narita, Natsumi Nakaoka, Marie Pierre Krafft, Daisuke Koyama

**Affiliations:** 1 Faculty of Science and Engineering, Doshisha University, 1-3 TataraMiyakodani, Kyotanabe, Kyoto 610-0321 Japan; 2 Institut Charles Sadron (CNRS), University of Strasbourg, 23 rue du Loess, 67034 Strasbourg, France

**Keywords:** Biomedical engineering, Phospholipids

## Abstract

Microbubbles have potential applications as drug and gene carriers, and drug release can be triggered by externally applied ultrasound irradiation while inside blood vessels. Desorption of molecules forming the microbubble shell can be observed under ultrasound irradiation of a single isolated microbubble, and the volume of desorbed molecules can be quantitatively estimated from the contact angle between the bubble and a glass plate. Microbubbles composed of a 1,2-dimyristoyl-*sn*-glycero-3-phosphocholine (DMPC) shell and a poorly-soluble gas are created. When the microbubbles are exposed to a pulsed ultrasound, the contact angles increase dramatically; the percentage of DMPC molecules desorbed from the bubble surface reaches 70%. Vibration of a single bubble in the radial direction is measured using a laser Doppler vibrometer. The relationship between the vibrational characteristics and the amount of molecular desorption reveals that a larger vibrational amplitude of the bubble around the resonance size induces a larger amount of molecular desorption. These results support the possibility of controlling molecular desorption with pulsed ultrasound.

## Introduction

In vascular drug therapies, side effects on healthy tissues are serious concerns, and drug delivery systems (DDSs) capable of local drug release have been developed to address this considerable problem. In DDSs using ultrasound, drug- or gene-containing microbubbles with a diameter of several microns are used as drug carriers, injected into blood vessels and transported by blood flow^1–5^. The microbubbles used for DDSs are modified chemically so that they can adsorb specifically to the target tissues, and ultrasound excitation can trigger the bubbles to oscillate and release the drug locally. Typical contrast agents used in medical ultrasound imaging are coated with phospholipids or proteins, and a perfluorocarbon gas that is poorly soluble in water is employed to prolong microbubble lifetime in blood vessels^6–10^. Understanding the behavior of microbubbles under ultrasound irradiation is important for medical ultrasound imaging techniques because the molecular film surrounding the gas core of bubbles largely affects their vibration and scattering signals, and understanding the bubble dynamics both experimentally and theoretically is an active area of research^11–16^. Microbubbles have been proposed for ultrasound-assisted DDSs based on ultrasound contrast agents, which means that the molecular shell plays an important role in drug release^11^. When a micrometer-sized bubble is subjected to a low-amplitude acoustic pressure field (under approximately 1 kPa in megahertz range ultrasound), it undergoes a spherical vibration (expansion and contraction) mode synchronized with the pressure change. As the sound pressure amplitude increases, nonspherical vibration modes are generated with surface waves propagating on the microbubble surface^17–21^, where the vibration mode mainly depends on the relationship between the sound pressure amplitude, driving frequency, and bubble size. A further increase in sound pressure amplitude induces collapse of the microbubble, with a microjet of the internal gas penetrating the molecular film because the bubble cannot maintain its shape due to the violent vibration^22^. In ultrasound DDSs, local drug delivery can be achieved by exploiting the collapse of microbubbles adsorbed on the target tissues, since the jet flow generated by the bubble collapse can penetrate the cell membrane and release the drug inside the cell through the small pores created (a process so-called “sonoporation”)^23–29^. However, van Wamel et al. reported that microbubble collapse is not mandatory for sonoporation. The transfer efficiency of drugs into the cells is indeed sufficiently improved in the absence of bubble collapse at smaller sound pressure amplitudes (hundreds of kPa). This implies that the pores are produced during the bubble contraction phase, releasing the drug into the cell in the expansion phase^29^. These results demonstrate that the quantification and control of molecular desorption from a bubble surface, induced by ultrasonication, are essential, not only to control the amount of drug but also to increase the safety of ultrasound DDSs, as lower sound pressure significantly decreases the risk of tissue damage.

The effects of a molecular film formed at a gas–liquid interface on the bubble vibration under ultrasound irradiation have been studied^12,30,31^ by classical ultrasound backscattering methods^32–34^ and optical observation using high-speed cameras^28,35–37^, and theoretical modelling allows us to quantitatively evaluate the viscoelasticity of the molecular film^38^. From the viewpoint of interface engineering, the adsorption and desorption dynamics of the molecular film before and after ultrasonication should be clarified. Kooiman et al. have investigated the distribution of molecules on a bubble surface with fluorescent lipids, using fluorescence microscopy under pulsed ultrasound^39^, evaluating the desorption of lipids from the bubble via changes in the optical intensity. In our previous work^40^, the adsorption and desorption of a surfactant from a single bubble were investigated by measuring the change in contact angle of the bubble on a glass plate. Although ultrasound-induced transient desorption of surfactants was successfully observed, the molecules desorbed from the bubble surface to the surrounding medium re-adsorbed immediately because the experiments were conducted using dispersions saturated in surfactant, meaning that the amounts of molecular desorption from the bubble surface could not be estimated quantitatively and this experimental condition was far from that in blood vessels. In addition, the lifetime of the microbubbles is also important for ultrasound imaging and DDS techniques, which depends on not only the molecular density on the bubble surface^41,42^ but also that of the surrounding medium. These facts mean that the molecular desorption from a single bubble under ultrasonication can be estimated quantitatively using a surrounding medium with no surfactant. To the best of our knowledge, there are few reports on the estimation of surfactants desorbed from single microbubbles induced by ultrasound, and determination of the temporal changes in the amounts of molecules on a bubble surface is important for vascular ultrasound DDSs. In this paper, we propose a method to measure the amounts of molecular desorption of 1,2-dimyristoyl-*sn*-glycero-3-phosphocholine (DMPC) on a bubble surface quantitatively from the changes measured in the contact angle. The relationship between vibrational amplitude and molecular desorption under ultrasound irradiation is also discussed.

## Experimental section

**Figure 1:**
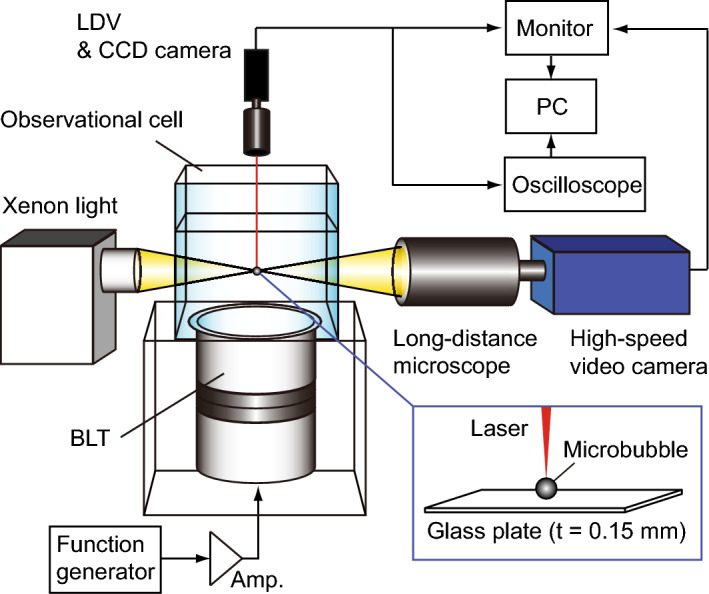
Observational setup for measuring the contact angle and vibration of a microbubble under ultrasound irradiation.

Surface tensiometry allows us to directly measure the surface tension of a single bubble through optical observation and evaluate adsorption kinetics on the bubble surface in the case of sub-millimeter-sized bubbles^43^. Here, the contact angle of single microbubbles on a glass plate in water is measured to estimate the amounts of molecules adsorbed on, and desorbed from, the bubble surface. 1,2-dimyristoyl-*sn*-glycero-3-phosphocholine (DMPC), purchased from NOF Corporation, Tokyo, Japan [CAS Registry No. 18194-24-6; *M*_w_ value of 677.9], is used as the surfactant for bubble formation, and a fluorocarbon-enriched gas (C_4_F_8_ 8% and N_2_ 92%, GL Sciences Corporation, Tokyo, Japan) is used as the internal gaseous phase of the microbubbles. Figure 1 shows the observation setup for the microbubbles, consisting of a xenon light source, a high-speed camera with a long-range microscope (HPV-1, Shimadzu, Kyoto, Japan), a laser Doppler vibrometer (LDV) (NLV2500, Polytech, Waldbronn, Germany) with an objective lens (M Plan Apo 20×, Mitutoyo, Kanagawa, Japan), and a transparent ultrasound cell (75 × 75 × 60 mm^3^). A bolt-clamped Langevin-type ultrasound piezoelectric transducer (Fuji Ceramics, Fujinomiya, Japan) with a resonance frequency of 38.8 kHz is attached at the bottom of the ultrasound cell, so that pulsed ultrasound can be transmitted to the cell filled with liquids. The sound pressure amplitude is measured with a calibrated homemade hydrophone. The ultrasound cell is filled with DMPC dispersions or pure water, and a glass plate with a thickness of 0.15 mm is immersed in the cell for the measurement of the contact angle. Control of the water level in the cell allows constant maintenance of the electric admittance of the ultrasound transducer and the sound pressure amplitude. In this study, we conduct two experiments. Fluorocarbon gas bubbles (without a DMPC shell) in DMPC dispersions with several concentrations (0.1 to 5 mM) are used in the first experiment, and bubbles coated with a DMPC film in pure water are used in the second. DMPC dispersions are used with concentrations ranging from 0.1 to 5 mM, prepared by dispersing DMPC in phosphate-buffered saline (PBS) (Fujifilm Wako Pure Chemical Corporation, Osaka, Japan) using a magnetic stirrer. Polyethylene glycol monostearate [PEG, CH_3_(CH_2_)_16_COO-(CH_2_CH_2_O)_2_H); Fujifilm Wako Pure Chemical Corporation, Osaka, Japan; CAS Registry No. 106-11-6; *M*_w_ value of 372.66] is added to the DMPC dispersions in consideration of future applications in clinical research (e.g. 57 mg of DMPC and 39 mg of PEG are added to 250 ml of PBS for 0.3 mM DMPC dispersion).

In the first experiment, the ultrasound cell is filled with the DMPC dispersions, and the naked fluorocarbon bubbles are fabricated directly onto the glass plate using a syringe with a microneedle. For the second experiment, microbubbles coated with a DMPC molecular film are fabricated using two connected syringes. For example, a syringe filled with 3 ml of 0.3 mM DMPC dispersion is connected to another syringe filled with 6 ml of fluorocarbon gas and shaken 30 times by hand to create microbubbles with a molecular film. The bubble dispersions are sonicated using an ultrasonic homogenizer (Q125, WakenBtech, Kyoto, Japan) for one minute at 20 kHz, with the vibrational displacement of 108 μm on the tip to form DMPC microbubbles with a radius of 20–440 μm. The DMPC microbubbles are attached on the glass plate in the ultrasound cell using a syringe. The glass plate does not disturb the acoustic field in the cell because the plate thickness (0.15 mm) is much smaller than the ultrasound wavelength (~ 39 mm. In fact, the sound pressure distributions in the cell with and without the glass plate were almost the same, which was confirmed by measurement using a hydrophone.). The ultrasound cell is filled with degassed water to prevent exchange of the gas between the inside of the bubble and the surrounding water through the DMPC molecular film.

**Figure 2:**
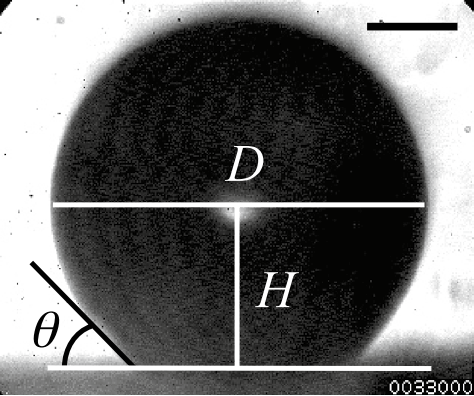
Photograph of a microbubble attached to the glass plate in the ultrasound cell. The scale bar indicates 100 μm.

The incident light is focused on the microbubbles on the glass plate, with the transmitted light received by the high-speed camera via the long-distance microscope, in order to measure the contact angle between bubble and glass. Figure 2 shows a representative photograph of a microbubble. The contact angle of a bubble on the glass plate *θ* can be expressed geometrically as

1 $$\theta = \cos^{ - 1} \left( \frac{2H}{D} \right),$$

where *H* is the distance from the center of the bubble to the surface of the glass plate, and *D* is the horizontal diameter of the bubble. The bubble wall (gas–liquid interface) is determined from the spatial gradient of the image brightness. It should be noted that microbubbles are observed with diameters of 20–440 μm, larger than the 1–5 μm bubbles used in clinics, because the image resolution of the high-speed camera is 1.69 μm/pixel. Considering the time constant of the desorption of DMPC molecules, the change in contact angle of the microbubble is measured every 30–120 s. Although the most common observational method for microbubble vibration^28,35–37^, microscopic observation using high-speed cameras requires higher image resolution compared with the vibrational displacement of microbubbles and cannot be used to measure a small vibrational amplitude under low sound pressure amplitude. By contrast, the LDV enables precise measurement of bubble vibration with a small amplitude at the nanometer level^15^. The sensor head of the LDV is set above the ultrasound cell, and the radial vibrational displacement amplitude of the microbubbles is measured by adjusting the focal point of the LDV to the top of the bubble (the spot size of the laser beam is 1.5 μm). The size of the microbubble is measured through a CCD camera installed in the sensor head (the image resolution is 1.02 μm/pixel). A sinusoidal pulsed signal for 50 cycles with a frequency of 38.8 kHz is input to the ultrasound transducer, and the maximum sound pressure amplitude at the attachment position of the microbubble is controlled to be 20 kPa. All experiments are conducted at 22 °C.

## Results and discussion

**Figure 3:**
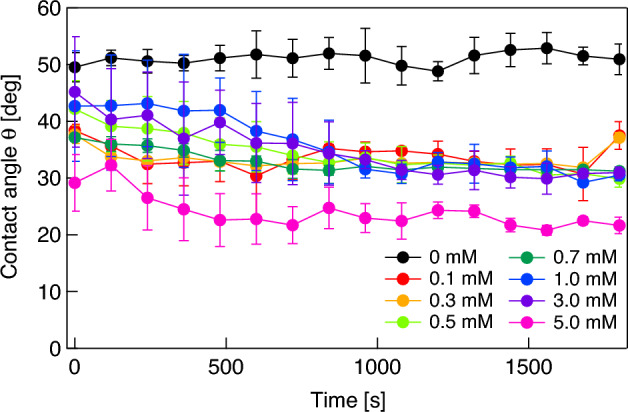
Changes in contact angle of a microbubble attached to the glass plate in water (0 mM) and in DMPC dispersions with concentrations ranging from 0.1 to 5.0 mM.

**Figure 4:**
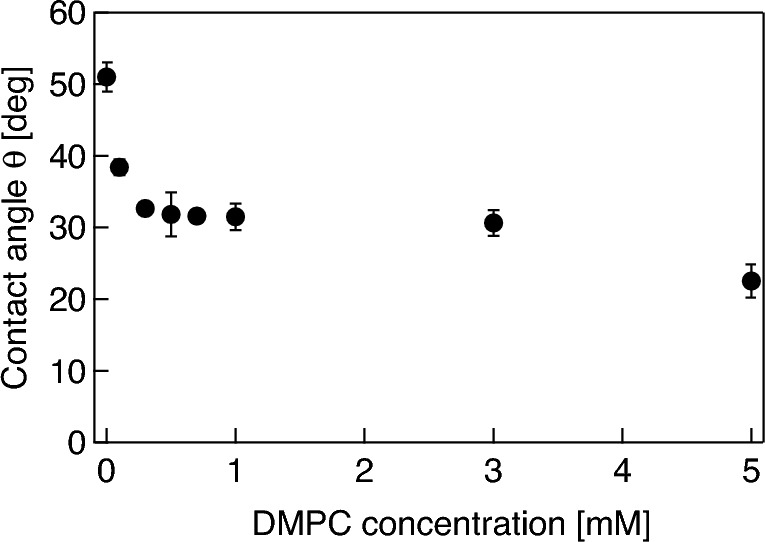
Relationship between the DMPC concentration in the dispersion and the contact angle of a microbubble attached to the glass plate.

In the first experiment, the DMPC molecules present in the solution begin to adsorb to the surface of naked fluorocarbon microbubbles attached on the glass plate, causing a decrease in the surface tension of microbubbles, and a consequent decrease in contact angle. Figure 3 shows representative results of temporal changes in the contact angle of a microbubble formed in the DMPC dispersion with concentrations ranging from 0 mM (water) to 5 mM. The plots and error bars represent average values and the standard deviations for three trials, respectively. Note that time *t* = 0 is arbitrary because some time is required to control the focal point of the camera on the bubble after the bubble is attached to the glass plate. Although the contact angle is determined by surface tension, which is almost independent of bubble size, the buoyancy force acting on the bubble distorts it from spherical, resulting in slight differences in contact angle. The contact angle of the bubbles decreases gradually with time for all the DMPC-containing dispersions. The changes in contact angle reach a steady state after approximately *t* = 1000 s for all concentrations, meaning that the adsorption of DMPC molecules on the bubble surface also reaches the steady state in each dispersion. Higher DMPC concentrations gave a smaller terminal value for the contact angle, indicating a lower surface tension. To estimate the molecular desorption from the bubble surface under ultrasonication, the relationship between the contact angle of the bubbles and the density of DMPC molecules adsorbed on the bubble surface is important. Figure 4 shows the relationship between the DMPC concentration and the contact angle of the microbubble in each saturation state. The contact angle changes largely under 0.3 mM and over 3 mM, while it does not change much in the 0.3–3 mM range, suggesting that the DMPC molecules forming a shell at the bubble surface undergo two successive phase transitions at 0.3 and 3 mM. Change in the surface pressure of other surfactants such as Pluronic F-68 measured by tensiometry show a similar trend^44^.

**Figure 5:**
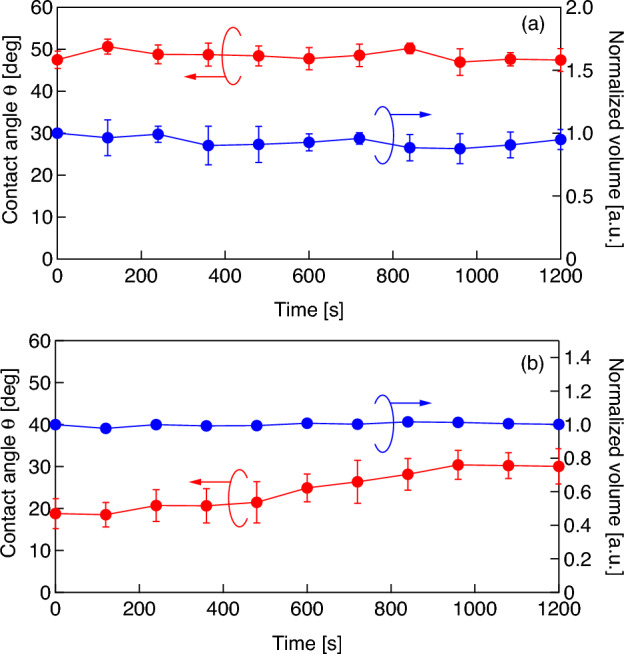
Changes in the contact angle (red) and volume (blue) of a microbubble (**a**) without and (**b**) with a DMPC molecular film in water with respect to time.

**Figure 6:**
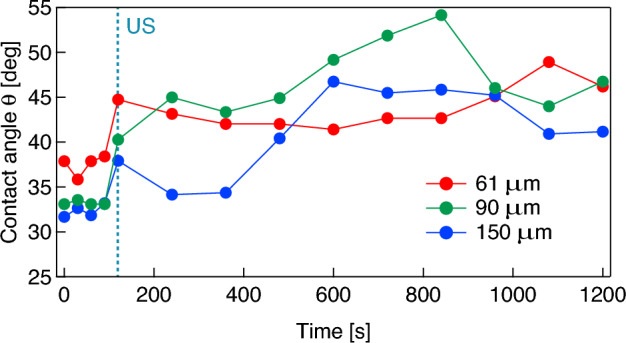
Changes in the contact angle of microbubbles attached to the glass plate (radii of 61 μm (red), 90 μm (green), and 150 μm (blue)) with respect to time. “US” at *t* = 120 s indicates the 50-cycle pulsed ultrasonication with 20 kPa at 38.8 kHz.

In the second experiment series, microbubbles coated with a DMPC molecular film are attached on the glass plate in pure water. Figure 5 shows the representative results of changes in the contact angle and volume of microbubbles (a) without and (b) with a DMPC molecular film; the initial bubble radii in Fig. 5a,b at *t* = 0 range from 147 to 226 μm. The plots and error bars represent average values and the standard deviations for three trials, respectively, and the right vertical axis indicates the volume of microbubbles normalized by the initial bubble radius at *t* = 0. In the case of the naked microbubble (Fig. 5a), the contact angle and volume are stable, meaning that the internal fluorocarbon gas of the bubble dissolves only little into the surrounding medium (degassed pure water). Strictly speaking, the bubble dissolves gradually with a long time constant because the Laplace pressure is low^45^. However, the contact angle for the DMPC-coated bubble increases gradually while its volume remains stable (Fig. 5b). These results imply that the DMPC molecules are desorbed gradually from the bubble surface because it has a higher molecular density than that of the surrounding medium. For the second experiment, microbubbles are created using a 0.3 mM DMPC dispersion because the gradient of the experimental curve shown in Fig. 4 is comparatively large under 0.3 mM, resulting a large change in contact angle induced by molecular desorption. Figure 6 shows changes in the contact angle of the DMPC bubbles of different three radii (61, 90, and 150 μm), with respect to time, when the bubbles are exposed to pulsed ultrasound in pure water. The bubbles are irradiated by a 50-cycle pulsed ultrasound with a sound pressure amplitude of 20 kPa at 38.8 kHz only once at *t* = 120 s, with *t* = 0 corresponding to the beginning of the observation. The contact angles of the bubbles are dramatically increased by the temporal pulsed ultrasound and continue to increase gradually after the ultrasonication in a way reminiscent of the trend illustrated in Fig. 5b.

**Figure 7:**
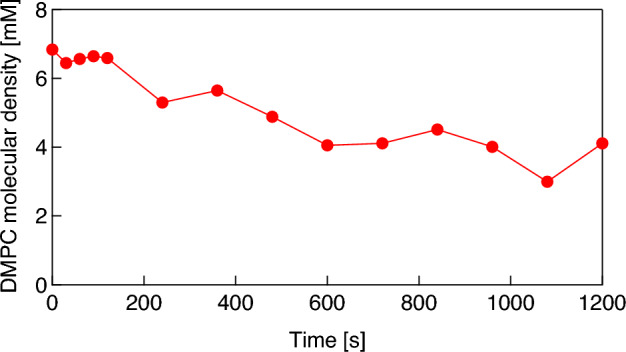
Change in the density of DMPC molecules adsorbed on the surface of a microbubble with an initial radius of 147 μm with respect to time.

**Figure 8:**
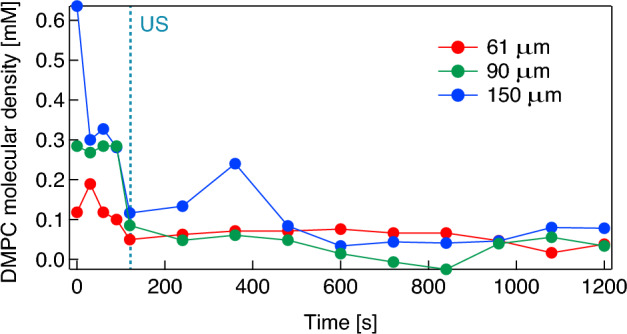
Changes in the density of DMPC molecules adsorbed on the surface of a microbubble with a radii of 61 μm (red), 90 μm (green), and 150 μm (blue) with respect to time. “US” at *t* = 120 s indicates the 50-cycle pulsed ultrasonication with 20 kPa at 38.8 kHz.

Assuming that the change in contact angle of the bubble induced by the desorption of DMPC molecules from the bubble surface is sufficiently rapid, that is, if the contact angle changes with the molecular desorption almost simultaneously, the temporal change in the density of DMPC molecules on the bubble surface can be estimated from the change in contact angle via the experimental result in the saturation states shown in Fig. 4. In addition, it should be noted that surface excesses (mol·m^−2^) are generally used to evaluate molecular density on a gas–liquid interface, and here we evaluate the molecular density on the bubble surface using the DMPC dispersion (mM). This is under the assumption that the amount of DMPC molecules adsorbed on the bubble surface is proportional to the molecular density of the DMPC dispersion under 0.3 mM. Figure 7 shows the change in the density of the DMPC molecules on the bubble surface in pure water, calculated from the result shown in Fig. 5b. From the initial value of 6.8 mM, the molecular density decreases to 4.1 mM at 1200 s with a time constant of 1014 s in the absence of ultrasonication. Figure 8 shows the change in the density of the DMPC molecules on the bubble surface for the three differently sized microbubbles under pulsed ultrasound, calculated from the result in Fig. 6. Note that the reason the initial molecular density before ultrasonication for the 61 μm bubble is approximately 0.1 mM, not 0.3 mM, is that preparation time is required to control the focal point of the camera on the bubble, and the molecular density on the bubble surface decreases gradually in water as described above. The molecular densities on the bubble surface are decreased significantly by ultrasonication; those for bubbles with radii of 61, 90, and 150 μm change from 0.10, 0.28, and 0.28 mM to 0.05, 0.09, and 0.12 mM, respectively. These results mean that significant portions of the DMPC molecules are desorbed from the bubble surface due to vibration at the gas–liquid interface induced by the pulsed ultrasound, and 50, 70, and 58% of the molecules are desorbed by one pulsed ultrasonication from the surfaces of the microbubbles with radii of 61, 90, and 150 μm, respectively. The decrease in the contact angle was observed by the high-speed camera at a shutter speed of 4000 frames/s immediately after pulsed ultrasonication, meaning the time constant of the molecular desorption was short and can be estimated to 2 ms at most. These results imply that the desorption is a major factor of the loss of phospholipid because this time constant is much shorter than those of other processes (tens of seconds)^45^. In addition, buckling phenomenon, wrinkles, or vesiculation in the DMPC shell were not observed by the high-speed camera under ultrasound irradiation with a small pressure amplitude (20 kPa). This also supports that desorption is the main mechanism for lipid loss (the same trend was observed by Kwan and Borden^45^ and Lozano and Longo^46^). In fact, only the spherical and linear vibration of microbubbles was observed by the camera and the LDV. Following ultrasound irradiation, the molecular density decreases continuously and gradually as shown by the results presented in Fig. 7.

**Figure 9:**
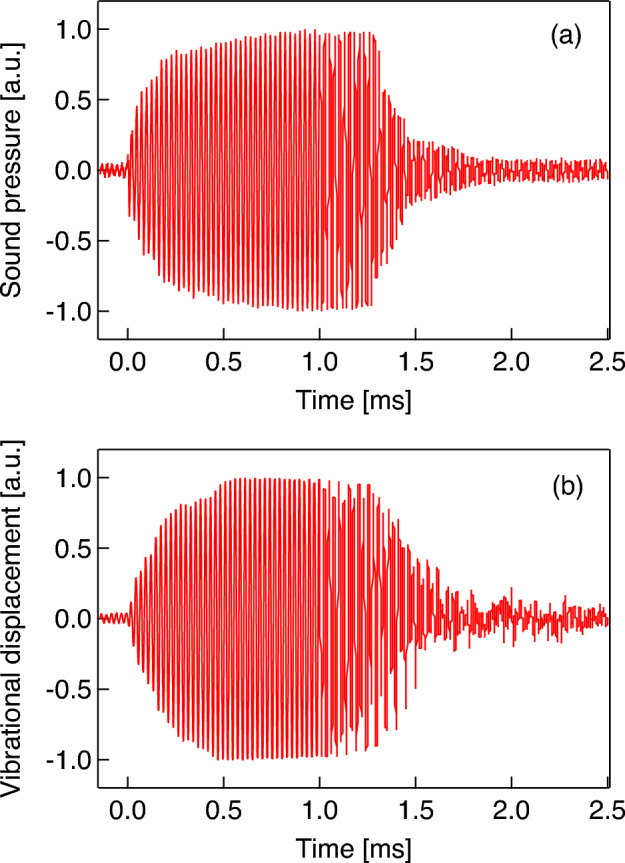
Waveforms of (**a**) the pulsed sound pressure in the ultrasound cell at 38.8 kHz and (**b**) the vibrational displacement of a microbubble with a radius of 115 μm measured by the LDV.

**Figure 10:**
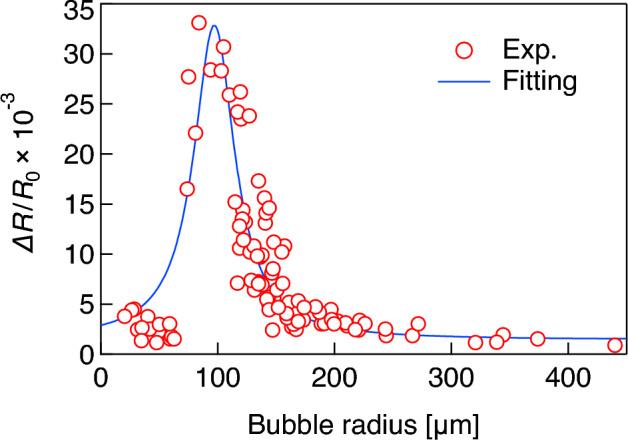
Relationships between the initial bubble radius *R*_0_ and the normalized vibrational displacement amplitude (*ΔR*/*R*_0_) at 38.8 kHz. The plots and solid line indicate experimental results for 99 samples and the fitting curve using a Lorentz function.

Figure 8 indicates that the amount of molecular desorption induced by ultrasonication is dependent on bubble size, meaning that the vibrational characteristics of microbubbles are key for controlling the drug release in DDSs. Figure 9 shows the representative waveforms of the sound pressure at 38.8 kHz in the ultrasound cell and the vibrational displacement of the bubble with a radius of 116 μm, measured by the LDV. A 50-cycle pulsed electric signal is input to the ultrasound transducer at *t* = 0, giving a maximum sound pressure amplitude of 20 kPa. Although both sound pressure and bubble vibration reach a steady state at approximately *t* = 0.5 ms (after approximately 20 cycles) in Fig. 9, the transient response of bubble vibration is dependent on bubble size^47^. For 99 microbubbles with radii ranging from 20 to 440 μm, the vibrational displacement amplitudes are measured under the same conditions (50-cycle pulsed ultrasound at 38.8 kHz with 20 kPa). Figure 10 shows the relationship between the initial bubble radius (i.e., the bubble radius just before ultrasonication) and the vibrational displacement (the so-called *resonance curve*), where the vertical axis indicates the vibration displacement amplitude in the steady state, *ΔR*, normalized by the initial bubble radius *R*_0_. The plots indicate values measured by the LDV, with the solid line showing a curve fitted with a Lorentz function^23^. The resonant bubble radius, in which the normalized vibrational displacement is maximized, is estimated to be 97 μm at 38.8 kHz from the fitting curve. The resonance size of microbubbles can be determined by two factors. The attachment of a microbubble on a rigid wall gives a 10% decrease in resonance radius at 39 kHz^23^ although a resonant bubble radius at 38.8 kHz in water is calculated to be 84.6 μm using a simplified linear model for bubble vibration under low sound pressure^48^. In contrast, the molecular film on the bubble surface generally increases the resonance size due to viscoelasticity^12^. From the fitting curve shown in Fig. 10, the normalized vibrational displacement amplitudes for microbubbles with radii of 61, 90, and 150 μm (shown in Figs. 6 and 8) can be estimated to be 0.01, 0.03, and 0.006, respectively. It should be noted that the experimental result shown in Fig. 10 displays a large variation because the vibration of a microbubble under ultrasound irradiation is essentially a nonlinear phenomenon and is influenced by adsorption and desorption of DMPC molecules on the bubble surface; the relative uncertainty was calculated to be 4.5%. Table 1 summarizes these results, including the desorption ratio of the DMPC molecules, implying that the desorption ratio by ultrasonication increases under the resonant condition, since molecular desorption is induced by the bubble vibration.

**Table 1:** Relationships between bubble radius, vibrational displacement, and desorption rate of DMPC molecules.

Bubble radius (μm)	Normalized vibrational displacement (*ΔR*/*R*_0_)	Desorption rate (%/pulsed ultrasonication)
61	0.01	50
90	0.03	70
150	0.006	58

In this study, bubbles with diameters of 20–440 μm were used. These bubbles are significantly larger than the microbubbles used in the clinic (1–5 μm). The choice of such large bubbles is due to the limited image resolution of the high-speed camera. The fact that the viscoelasticity of lipid-shelled microbubbles changes with the bubble size^44,49^ implies that the curvature of the gas–liquid interface (bubble surface) may also impact the vibrational characteristics and the lipid desorption rates (intermolecular forces in the lipid shell are dependent on the curvature^50^). The bubble curvature may also impact bubble stabilization^51^. Several researchers reported that the composition of lipids adsorbed on microbubbles impact the dissolution process^45^ (for example, long-chain lipids strongly increase the lifetime of a microbubble^46^). The acoustic response of microbubbles is also affected by the lipids adsorbed on the bubble since the viscoelasticity of the lipid shell changes^52^. These facts mean that addition of several components on the bubble surface would significantly impact the desorption rates because the vibrational characteristics of the bubbles would be changed. In addition, considering the clinical use of microbubbles as drug/gene carriers, their surrounding medium would impact the desorption rates. If the microbubbles are injected into the blood circulation where nitrogen, oxygen, and carbon dioxide are present, both influx and efflux of the core gas and dissolved gas in blood would occur, resulting in changes in the bubble size^53^, as well as changes in the desorption rates with time. In addition, the surrounding environment of microbubbles in blood would affect the desorption rate. For, example, an increase of the viscosity of the surrounding fluid would decrease the vibrational displacement amplitude of the bubble and the desorption rate.

## Conclusions

We propose a method to observe desorption of the shell-forming phospholipid (DMPC) from a single microbubble under pulsed ultrasound. Microbubbles coated with a DMPC molecular film are fabricated, and optically observed using system composed of a high-speed camera, an LDV, and an ultrasound cell to investigate the relationship between molecular desorption and the vibrational characteristics of microbubbles. Change in the molecular density on the bubble surface is estimated from the change in contact angle of the bubble attached on a glass plate. Microbubbles under the resonance condition release significant amounts of surface-adsorbed molecules to the surrounding media: 70% of DMPC molecules are desorbed from the bubble surface by a 50-cycle ultrasound pulse at 38.8 kHz with 20 kPa. The desorption by ultrasonication is not limited to DMPC, and the same effect was observed in the case with other surfactants such as Pluronic F_68_. In addition, the same phenomenon is probably observed with smaller microbubbles used as ultrasound contrast agents because the curvature is too large to influence the dynamics of the surfactants at the gas/water interface even in this case^54^. For controlled drug release, we intend to clarify the relationship between the ultrasound frequency, sound pressure amplitude, and the quantity of molecular desorption in our future work.

## Data Availability

The datasets used and/or analysed during the current study available from the corresponding author on reasonable request.
